# Acoustic Analysis of Swallowing of an Experimental Meal of Three Food Textures: A Comparative Aging Study

**DOI:** 10.1007/s00455-023-10629-3

**Published:** 2023-11-18

**Authors:** Jean Baqué, Océane Huret, Pierre Rayneau, Marianne Schleich, Sylvain Morinière

**Affiliations:** 1 ENT and Head and Neck Surgery, University Hospital of Tours, 2 Boulevard Tonnelé , 37044 Tours, France; 2 Francois‐Rabelais University of Tours, University Hospital of Tours, 10 Boulevard Tonnelé, 37032 Tours, France

**Keywords:** Deglutition, Presbyphagia, Swallowing sounds, Acoustic analysis, Aging

## Abstract

Swallowing disorders in the elderly represent a public health problem, their detections are a medico-economic issue. The acoustic analysis of swallowing has the advantages of being non-invasive with no radiation, compared to videofluoroscopy or fiberoptic swallowing assessments. Acoustic analysis of swallowing has been tested in many studies but only on small food boluses. The aim of this study was to compare the acoustic swallowing parameters of two groups of healthy subjects, before and after 70 years old, during the intake of a series of 3 food textures. A laryngophone was used to record the pharyngeal phase of swallowing. The experimental meal was composed of 100 ml of mashed potatoes, 100 ml of water, and 100 ml of yogurt. Group 1 (50–70 years old) comprised 21 subjects and group 2 (over 70 years old) 23 subjects. Acoustic parameters analyzed were the number of swallows, average duration of swallowing, average duration of inter-swallowing, meal duration, and the average frequency of swallowing per minute. These parameters for groups 1 and 2 were compared. The average duration of inter-swallowing and the meal duration were significantly higher in the older group (*p* < 0.001), with a mean duration of inter-swallowing that was 2.4 s longer than the younger group. The average swallowing frequency per minute was higher in the younger group (11.3 vs 7.9; *p* < 0.001). This study demonstrated that acoustic analysis of an experimental meal of three food textures generated usable data on swallowing. In the over 70 age group, there was a decrease in swallowing frequency, indicating a slowdown in food intake. A reduced swallowing frequency could become a criteria to assess presbyphagia.

**Supplementary Information:**

The online version contains supplementary material available at 10.1007/s00455-023-10629-3.

## Introduction

With aging, many changes are involved, such as reduced muscle mass, lower saliva production, reduced sense of taste and smell [1–3], and less coordination between swallowing and respiratory functions [4]. These age-related physiological changes in the swallowing mechanism correspond to presbyphagia and are not considered pathological [5]. Presbyphagia can be responsible for changes in eating habits, with the implementation of compensatory strategies by the elderly, such as reducing the quantities ingested [6]. However, there is no consensus on the age at which presbyphagia can be considered, which varies from 60 to 75 depending on the study [5, 7, 8]. Combined with other co-morbidities, swallowing functions may be impaired beyond physiological deterioration, with the patient entering the framework of dysphagia.

The gold standard methods to assess swallowing are videofluoroscopy (VFS) and fiberoptic endoscopic evaluation of swallowing (FEES) [9]. These techniques have the disadvantages of using radiation and of being invasive for the patients [10]. With the development of sensors positioned on the head or the neck, the assessment of swallowing tends to be less invasive. Some teams have conducted studies on swallowing with surface electromyography and accelerometry to record suprahyoid muscles and laryngeal activation [11]. Others used bend sensors to detect laryngeal movements [12, 13]. Acoustic analysis of swallowing sounds is another non-invasive method in full expansion, consisting in placing microphones on the neck and analyzing the acoustic signal. Many studies have evaluated this method and demonstrated it was possible to recognize a swallow on an audio recording and to obtain useful parameters, such as the duration of a swallow [14, 15]. Other studies have already carried out analyses on swallowing acoustics, with a comparison between groups of patients according to age [15, 16]. Their protocol proposed the ingestion of few boluses of food items or water, ranging from 5 to 15 cc. However, up to date, there are not any studies that have acoustically analyzed swallowing according to age, over a meal with several textures.

Conducting a study with presbyphagic patients requires choosing a threshold age. The lack of consensus on this subject can be compensated by a review of the literature. Labeit et al. [5] used 70 as a threshold age to evaluate presbyphagia in the elderly, when Jardine et al. took the same age threshold to distinguish young and older healthy adults in their study on the onset of swallowing disorders [17]. This period around the age of 70 is indeed at risk of developing pathological swallowing disorders, due to the increased incidence of dementia and stroke [18–20]. These pathologies, recognized causes of dysphagia [21, 22], can decompensate a healthy but fragile swallowing function in a presbyphagic patient. The age threshold of 70 therefore seems consistent for studying presbyphagia.

By analyzing the swallowing of different textures according to age, it is possible to detect signs of the onset of swallowing disorders. The identification of differences, between a moderate age group and an elderly group, would document the aging of swallowing function with concrete and quantified values. These differences could be used to identify indicators of a deterioration in swallowing function. These indicators could then be applied to detect onset of swallowing disorders and select patients who would benefit from speech therapy. Management of these early swallowing disorders should prevent or delay the onset of presbyphagia or even dysphagia. The aim of this study was to compare the acoustic swallowing parameters of two groups of healthy subjects, before and after 70 years old, over an experimental meal of three food textures. This study also serves to enlarge the database on acoustic swallowing analysis, providing results that can be compared with future studies in this area.

## Materials and Methods

### Subjects

In this prospective study, the protocol established two different age groups, group 1 (50–70 years old) and group 2 (over 70 years old). The study protocol was approved by our hospital’s ethics committee, consent was obtained from all subjects before participation in the study. Twenty-five subjects were included in each group, between January 2021 and December 2022. Subjects in group 1 were healthy volunteers, without any pathology that could impact swallowing. Subjects in group 2 were patients hospitalized in the geriatric department. In the absence of a consensus on the age of presbyphagia, the 70-year age limit was chosen on the basis of data in the literature, and the minimum age required for geriatric hospitalization in our center, which is 70. Exclusion criteria for both groups were the presence of swallowing disorders and pathologies that can influence swallowing (neurological disorders, respiratory condition), history of cervical surgery, cognitive disorders, and allergy to the products used for the tests.

### Placement of the Subjects

**Fig.1:**
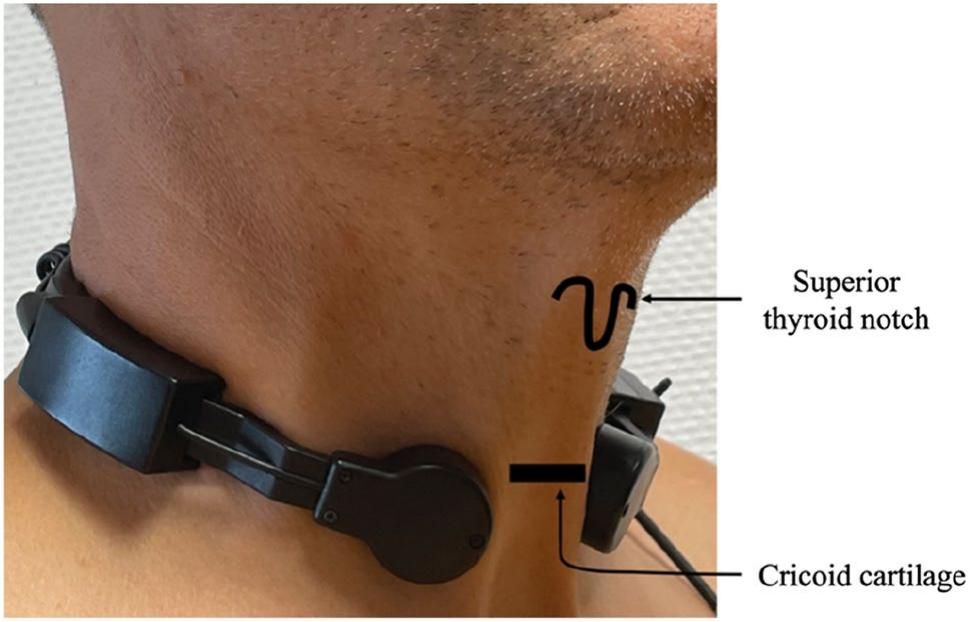
Laryngophone in place on a subject’s neck

Recordings took place in a quiet room. We used a laryngophone Nauzer^®^ (ref. PLX 300 K) for the acoustic recordings of the pharyngeal phase of swallowing. Two microphones were placed on each side of the neck in front of the trachea, just under the cricoid cartilage (Fig. 1). This is an optimal area for the detection of swallowing sounds [23]. Subjects needed to have a clear neck, no shirt collar, and no jewelry, with hair tied. The laryngophone was connected via an adapter to a tablet Samsung^®^ Galaxy Table 3, used to collect acoustic data. The last food intake had to be at least 3 h before the experimental meal for the study.

**Fig. 2:**
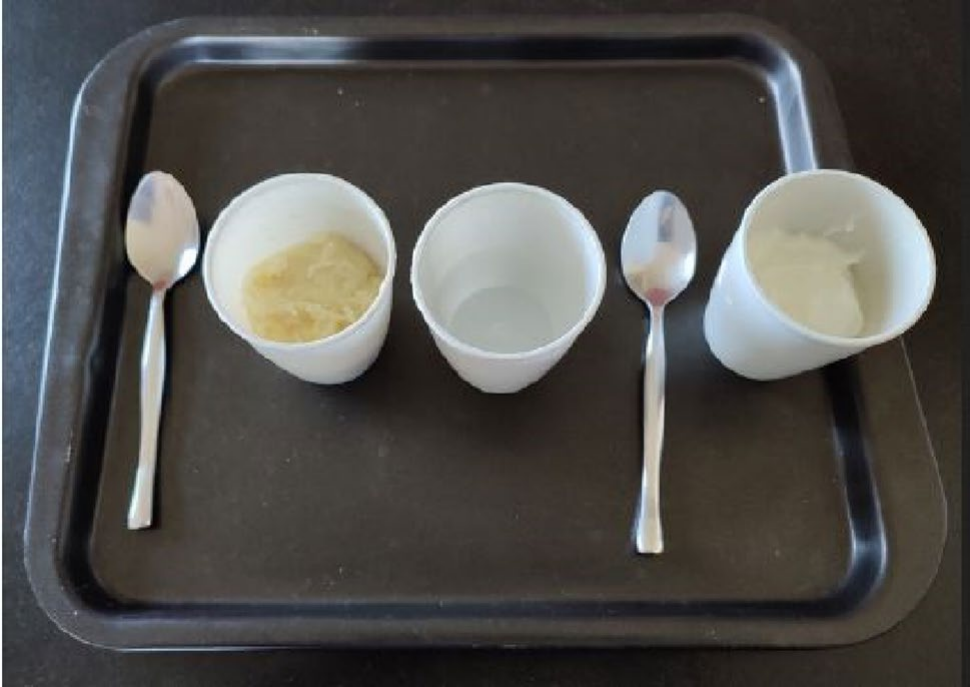
Experimental meal with its 3 components: mashed potatoes, water, and yogurt

To avoid a bias related to the dental status of the subjects, the selected meal components did not need to be chewed. The experimental meal was composed of 3 elements with different viscosity: plain water at room temperature, unsweetened yogurt (Danone^®^), and mashed potatoes (Mouseline^®^, 125 g in 250 ml of milk and 500 ml of water at 30 °C). The dynamic viscosity measured by the Brookfield method was 1 mPa.s, 300 mPa.s, and 50,000 mPa.s, respectively. The measurement of 100 mL of mashed potatoes, water, and yogurt was done using a measuring glass. The different substances were provided in 3 identical opaque plastic glasses, and two metal spoons were available to eat the mashed potatoes and the yogurt (Fig. 2). This food association (puree-water-yogurt) has already been used in previous works studying the swallowing function [24, 25].

The subjects were in a sitting position facing a table, and were asked to eat the experimental meal with the laryngophone in place on the neck: 100 mL of mashed potatoes, 100 mL of water, and then 100 mL of unsweetened yogurt. They were requested to eat at their usual pace. Due to the specificity of the software, which needs to be restarted for each new recording, we have left an identical delay of one minute between each meal component for all subjects.

### Acoustic Acquisition

The laryngophone was connected by an adapter to the tablet. We recorded the subject’s information: name, surname, sex, and date of birth. Then, we manually started and ended the recording for each of the mashed potatoes, water, and yogurt components. We obtained 3 audio files per subject, one per texture tested (mashed potatoes, water, yogurt) in a “.wav” audio file.

### Acoustic Analysis

**Fig. 3:**
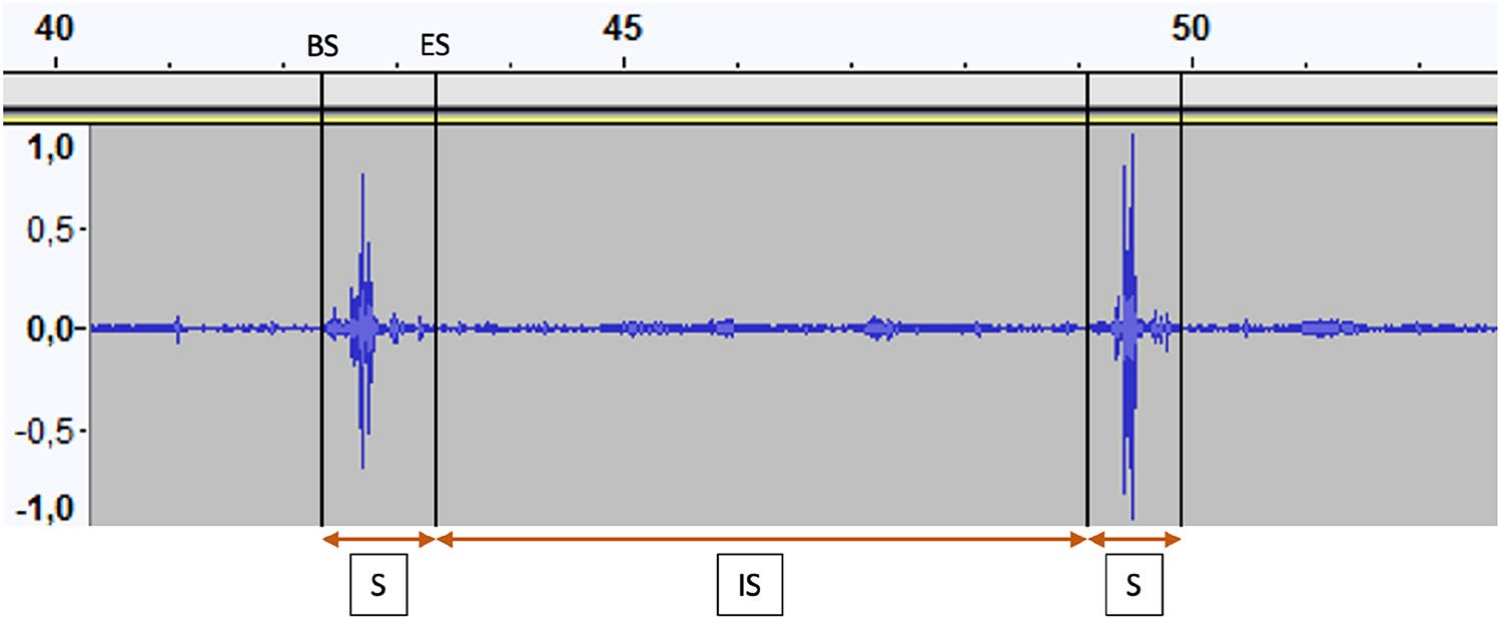
Part of an audio recording on the Audacity^®^ software, showing 2 swallows. *S* swallow, *IS* inter-swallow, *BS* cursor of beginning of swallowing, *ES* cursor of ending of swallowing

All audio recordings were transferred to a computer and analyzed on the Audacity^®^ software, which is a digital audio editing software (Fig. 3). Analyses on this software were performed by two different examiners who had been trained to use the software to analyze acoustic signal of swallowing.

To recognize swallows on audio recordings, the examiners relied on the work of Morinière et al. [14, 26] on the sound components of swallowing. Each recording was treated in the following way: a cursor was placed at the beginning and end of each swallow and the duration of each swallow and inter-swallow was then measured. The swallows identified on the recordings corresponded to the pharyngeal phase of swallowing.

**Fig. 4:**
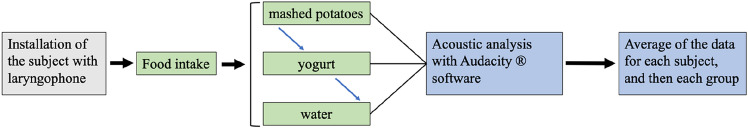
Study design

For each subject, different swallowing parameters were studied. The number of swallows (NS) was counted in each audio recording and then summed to obtain the NS of the experimental meal. The duration of each swallow was measured, taking the duration between the cursors at the beginning and at the end of the swallow. For all subjects, the average duration of swallowing (ADS, corresponding the pharyngeal phase of swallowing) for each audio recording, and for the experimental meal, was then calculated. The duration of each inter-swallow was also measured; it corresponded to the duration between the cursor at the end of a swallow and the cursor at the beginning of the next swallow. For all subjects, the average duration of inter-swallowing (ADIS) was then calculated for each audio recording and for the experimental meal. The duration of the meal itself (meal duration: MD) was measured for each meal component, by taking the duration between the cursor at the beginning of the first swallow and the cursor at the end of the last swallow. By adding the sum of the MD of the mashed potatoes, yogurt, and water, we found the MD of the experimental meal (Fig. 4).

Finally, the average swallowing frequency (ASF), corresponding to the average number of swallows per minute, was calculated from the NS and the MD for the experimental meal.

We have thus obtained for all subjects the NS, ADS, ADIS, and MD, for each meal components and for the experimental meal. We also obtained the ASF for the experimental meal. By averaging these parameters for the subjects in each group, the swallowing parameters of group 1 and 2 were calculated and compared.

### Statistics

All time values for this study were measured in milliseconds (ms), and statistical analyses were performed using the software GraphPad Prism version 5. We verified that the collected values were distributed according to a normal distribution with a Shapiro–Wilk test. In case of normal distribution, a Student’s t test was used to compare the data of the two groups. In case of non-normal distribution, a Mann–Whitney test was performed to compare the data of the two groups. The differences were considered significant when *p* value was less than 0.05, in a two-tailed statistical analysis.

## Results

Six subjects were excluded from the data analysis. Two could not finish the yogurt, one had dyspnea during the test disturbing the acoustic recording, one due to computer data loss, one because the collar was too small for his neck, and one because the laryngophone has been incorrectly connected to the tablet. We were therefore able to analyze data from 44 subjects (Table 1).

**Table 1:** Group characteristics

	Group 1	Group 2	*p* value
Number of subjects	21	23	
Women/Men	11/10	11/12	0.762
Mean age in years	58	87	< 0.001*

The difference in age was assessed with independent *t* test and the female sex difference with *χ*^2^ test.

The distribution type of the data was checked before making comparisons between the two groups. The series of values collected in our study followed a normal distribution, except for the following: NS for water, NS for yogurt, NS over the entire experimental meal, ADIS for water, ADIS for yogurt, and MD for water.

### Comparison of groups 1 and 2 for Each Meal Component (Table 2)

**Table 2:** Swallowing parameters with significative difference for each meal component (mean values and standard deviation)

	Group 1	Group 2	*p* value
ADIS mp (ms)	7 841 (± 2 494)	10 401 (± 3 536)	0.005*
ADIS y (ms)	5 353 (± 1 564)	8 390 (± 1 981)	< 0.001*
ADIS w (ms)	2 749 (± 2 087)	4 923 (± 3 011)	0.009*
MD mp (ms)	75 926 (± 20 725)	100 634 (± 42 801)	0.021*
MD y (ms)	62 732 (± 27 389)	101 649 (± 41 173)	< 0.001*
MD w (ms)	14 445 (± 10 458)	36 800 (± 29 002)	0.002*

*ADIS* average duration of inter-swallowing, *MD* meal duration, *mp* mashed potatoes, *y* yogurt, *w* water, *ms* milliseconds

*Results with statistically significant difference

No significant difference was found between the two groups for the NS and ADS, for each of the 3 meal components.

The ADIS was different between the two groups for the 3 meal components, longer in group 2 (mashed potatoes: *p* = 0.005, yogurt: *p* < 0.001, water: *p* = 0.009). This difference was also found in the MD, which was increased for each meal component in group 2 (mashed potatoes: *p* = 0.021, yogurt: *p* < 0.001, water: *p* = 0.002).

### Comparison of Groups 1 and 2 for the Entire Experimental Meal (Table 3)

**Table 3:** Comparison of swallowing parameters for the entire experimental meal (mean values and standard deviation)

	Group 1	Group 2	*p* value
NS	27.4 (± 5.0)	28.5 (± 7.3)	0.502
ASD (ms)	644 (± 94)	706 (± 144)	0.101
ADIS (ms)	5 704 (± 1 454)	8 103 (± 2 046)	< 0.001*
MD (ms)	153 103 (± 47 124)	239 082 (± 96 753)	< 0.001*
ASF	11.3 (± 2.5)	7.9 (± 2.2)	< 0.001*

*NS* number of swallows, *ADS* average duration of swallowing, *ADIS* average duration of inter-swallowing, *MD* meal duration, *ASF* average swallowing frequency, *ms* milliseconds

*Results with statistically significant difference

There was no significant difference between the two groups in the NS (*p* = 0.570) for the entire experimental meal. The ADS, corresponding to the pharyngeal phase of swallowing, was 62 ms longer in group 2, but this increase was not significant (*p* = 0.101).

There was a significant increase in the MD in group 2 (*p* < 0.001), which was also observed for the ADIS (*p* < 0.001). The experimental meal was longer in group 2 on average by 1 min 26 s. The increase in MD was due to the increase in inter-swallow duration: the time between swallows was on average 2.4 s longer in group 2, while the ADS was not different between the two groups.

ASF was 11.3 (min = 6.6–max = 15.6) swallows per minute in group 1 and 7.9 (min = 5.2–max = 12.3) in group 2, being significantly lower in the elderly group (*p* < 0.001).

All data generated or analyzed during this study are included in this published article [and its supplementary information files].

## Discussion

Our study demonstrated that it was possible to acoustically analyze swallowing over an experimental meal of three food textures, obtaining different information such as the number of swallows or the average duration of the pharyngeal phase. The significant temporal differences between the two groups did not actually correspond to the pharyngeal phase of swallowing, but to the elements surrounding it during the inter-swallowing phases. A difference according to age was observed in the inter-swallowing duration and the meal duration, longer in the older group. In the younger group, a higher swallowing frequency compared to the older group was found. Age had no influence on the quantity per bite, the meal was completely eaten by all subjects, and there was no difference in NS between the two groups.

We found in this study that the frequency of swallowing during feeding decreased significantly in the older group. A similar result was found for spontaneous swallowing by Tanaka et al. [27], who demonstrated that swallowing frequency in daily life decreased in the elderly. The concordance of these results, showing a decrease in swallowing frequency during and outside mealtimes in the elderly, suggests that swallowing frequency is a reliable indicator for assessing swallowing in this population.

No age-related difference in the duration of pharyngeal phase was found in this study, compared to other studies. Im et al. [28] managed to show with VFS a significantly longer pharyngeal transit duration in elderly subjects. Pongpipatpaiboon et al. [29] also found longer pharyngeal phase in older adults using kinematic analysis on scanners. However, the average age of the young subject group in these studies was 26.6 and 32. Our results only show a tendency to a longer pharyngeal phase in the group 2, a difference may have been found if group 1 had been composed of younger subjects.

We observed that the significative increase in MD in group 2 was due to longer intervals between each swallow. This may be caused partially by the decreased speed of motor functions with age, slowing the use of cutlery and transfers of food into the mouth [30]. It was also probably due to a longer oral phase that precedes the pharyngeal phase. Christmas et al. [31] observed a decreased lingual strength and pressure with age and Affoo et al. [2] a reduced salivary flow, such factors making it more difficult for the food bolus preparation in the mouth. However, the oral phase could not be studied and the laryngophone is not able to detect the mastication sounds. The impossibility to distinguish the oral phase from the phase of food transfer to the mouth, during the inter-swallowing periods, represents a limitation of our study. We could not prove that oral phase was longer in the older group. Another limitation of our study is the non-consideration of a potential order effect, as subjects had to eat the 3 components of the experimental meal in the same order and without mixing them. While in real-life situations it is common to drink water throughout a meal, we did not allow the subjects to drink while eating mashed potatoes or yoghurt to enable segmentation of the 3 components of the meal. It seemed impossible to carry out a statistical analysis on each component of the meal, if they were mixed together.

Our experimental meal was only composed of soft or liquid components to avoid chewing difficulties in the older group; however, the daily diet of an adult is considerably more diversified. The consistency [28, 32] or viscosity [33] of food bolus has been shown to influence the duration of swallowing. Using products with textures harder to swallow, such as crackers or meat, could have unmasked a difference in the duration of pharyngeal phase between the two different age groups in this study. Increasing the quantities of each meal to improve the statistical power of this study does not seem possible either. Too much food would prevent many elderly subjects from finishing the meal, which was already the case for 2 subjects in this study. However, with the ingestion of 3 food items, this protocol offers an advantage and a new approach compared with previous studies using only low-volume boluses. Analysis over this experimental meal provides a large number of swallows, which can take into account variations in swallowing duration or rhythm as the meal progresses. These variations could not have been detected by analyzing just a few boluses. It was therefore important to carry out this study on an experimental meal comprising several food textures, in order to detect anomalies that appear after repeated swallows. This analysis on a set of foods is also closer to reality, as it is not pre-calibrated. Subjects are free to take as much or as little as they like with their cutlery and decide how many “boluses” they need to eat the quantity of food presented to them.

Cervical acoustic analysis has the advantages of being non-invasive, with no radiation, and of analyzing any type of food (which does not need to be radio opaque). This method can be carried out in any location, from the patient’s hospital room to a nursing home. These advantages are balanced by moderate inter-rater reliability [34], implying that the acoustic analysis should preferably be performed by the same examiner. This reliability must be validated on a large scale to use acoustic analysis of swallowing in current practice with patients. The reliability of cervical acoustic analysis of swallowing was previously studied by Jaghbeer et al. [34], who found good intra-rater reliability. Bergström et al. [35] managed to increase the reliability of acoustic analysis of swallowing with specific training. They set up a structured 2-day training course on cervical auscultation for 39 speech-language pathologists, finding a significant improvement in sound analysis and intra-rater reliability after the training. The need for specific training and the limited inter-rater reliability are still a drawback to the use of acoustic analysis. In addition to this disadvantage, only one part of the swallowing process is studied, cervical acoustic can analyze the pharyngeal phase, but not the oral and esophageal phases.

Our results showed, using acoustic swallow analysis, a delay between each swallow which increased in subjects aged over 70 compared with those aged between 50 and 70. In the older group, there is a decrease in swallowing frequency, indicating a slowdown in food intake. These results are a reminder that the study of swallowing should not be reduced to the act of swallowing, but should be conceived as a complex function involving interconnected processes, ranging from the use of cutlery to the arrival of the food bolus in the esophagus. A reduced swallowing frequency during feeding could thus become a criteria for assessing presbyphagia.

Further studies, using the same type of protocol with pathological subjects, will provide a continuum of data between healthy subjects, presbyphagic subjects, and dysphagic subjects. The result would be the creation of a score, based on swallowing frequency that could be used in clinical practice. Depending on the severity of their score, tested subjects would be referred for speech therapy or consultation with a swallowing physician. Automatic analysis of swallowing sounds, based on algorithms, has shown promising results in several studies [25, 36, 37]. Our long-term goal is to automatically obtain the swallowing frequency of the tested subjects, using an algorithm which analyzes the sounds of swallowing. The result would be a rapid, non-invasive method for indicating an individual’s swallowing frequency, usable on a large scale, guiding patients’ care pathways according to their results.

### Supplementary Information

Below is the link to the electronic supplementary material.

Supplementary file1 (XLSX 18 KB)

## Data Availability

All data generated or analyzed during this study are included in this published article [and its supplementary information files].

## References

[1] Azzolino D, Damanti S, Bertagnoli L, Lucchi T, Cesari M (2019). Sarcopenia and swallowing disorders in older people. Aging Clin Exp Res.

[2] Affoo RH, Foley N, Garrick R, Siqueira WL, Martin RE (2015). Meta-analysis of salivary flow rates in young and older adults. J Am Geriatr Soc.

[3] Braun T, Doerr JM, Peters L, Viard M, Reuter I, Prosiegel M (2022). Age-related changes in oral sensitivity, taste and smell. Sci Rep.

[4] Wang CM, Chen JY, Chuang CC, Tseng WC, Wong AMK, Pei YC (2015). Aging-related changes in swallowing, and in the coordination of swallowing and respiration determined by novel non-invasive measurement techniques. Geriatr Gerontol Int.

[5] Labeit B, Muhle P, von Itter J, Slavik J, Wollbrink A, Sporns P (2022). Clinical determinants and neural correlates of presbyphagia in community-dwelling older adults. Front Aging Neurosci.

[6] Namasivayam-MacDonald AM, Riquelme LF (2019). Presbyphagia to dysphagia: multiple perspectives and strategies for quality care of older adults. Semin Speech Lang.

[7] de Lima Alvarenga EH, Dall’Oglio GP, Murano EZ, Abrahão M (2018). Continuum theory: presbyphagia to dysphagia? Functional assessment of swallowing in the elderly. Eur Arch Otorhinolaryngol.

[8] Nakajima J, Karaho T, Kawahara K, Hayashi Y, Nakamura M, Matsuura N (2022). Latent changes in the pharyngeal stage of swallowing in non-aspirating older adults. Eur Geriatr Med.

[9] Wirth R, Dziewas R, Beck AM, Clavé P, Hamdy S, Heppner HJ (2016). Oropharyngeal dysphagia in older persons – from pathophysiology to adequate intervention: a review and summary of an international expert meeting. Clin Interv Aging.

[10] Hwang SH, Park CS, Kim BG, Cho JH, Kang JM (2015). Topical anesthetic preparations for rigid and flexible endoscopy: a meta-analysis. Eur Arch Oto-Rhino-Laryngol Off J Eur Fed Oto-Rhino-Laryngol Soc EUFOS Affil Ger Soc Oto-Rhino-Laryngol - Head Neck Surg.

[11] Alvarez-Larruy M, Tomsen N, Guanyabens N, Palomeras E, Clavé P, Nascimento W (2023). Spontaneous swallowing frequency in post-stroke patients with and without oropharyngeal dysphagia: an observational study. Dysphagia.

[12] Li Q, Hori K, Minagi Y, Ono T, Chen YJ, Kondo J (2013). Development of a system to monitor laryngeal movement during swallowing using a bend sensor. PLoS One.

[13] Murakami K, Minagi Y, Hori K, Uehara F, Salazar SE, Inoue M (2020). Evaluation of hyoid movement during swallowing using a bend sensor. J Oral Rehabil.

[14] Morinière S, Boiron M, Alison D, Makris P, Beutter P (2008). Origin of the sound components during pharyngeal swallowing in normal subjects. Dysphagia.

[15] Youmans SR, Stierwalt JAG (2011). Normal swallowing acoustics across age, gender, bolus viscosity, and bolus volume. Dysphagia.

[16] Cichero JAY, Murdoch BE (2002). Acoustic signature of the normal swallow: characterization by age, gender, and bolus volume. Ann Otol Rhinol Laryngol.

[17] Jardine M, Miles A, Allen J (2018). Dysphagia onset in older adults during unrelated hospital admission: quantitative videofluoroscopic measures. Geriatrics.

[18] Hendrie HC (1998). Epidemiology of dementia and Alzheimer’s disease. Am J Geriatr Psychiatry Off J Am Assoc Geriatr Psychiatry.

[19] Plassman BL, Langa KM, Fisher GG, Heeringa SG, Weir DR, Ofstedal MB (2007). Prevalence of Dementia in the United States: the aging, demographics, and memory study. Neuroepidemiology.

[20] Feigin VL, Forouzanfar MH, Krishnamurthi R, Mensah GA, Connor M, Bennett DA (2014). Global and regional burden of stroke during 1990–2010: findings from the global burden of disease study 2010. Lancet.

[21] Takizawa C, Gemmell E, Kenworthy J, Speyer R (2016). A Systematic review of the prevalence of Oropharyngeal Dysphagia in stroke, Parkinson’s disease, Alzheimer’s disease, head injury, and pneumonia. Dysphagia.

[22] Espinosa-Val MC, Martín-Martínez A, Graupera M, Arias O, Elvira A, Cabré M (2020). Prevalence, risk factors, and complications of oropharyngeal dysphagia in older patients with dementia. Nutrients.

[23] Takahashi K, Groher ME, Michi K (1994). Methodology for detecting swallowing sounds. Dysphagia.

[24] Hammoudi K, Boiron M, Hernandez N, Bobillier C, Morinière S (2014). Acoustic study of pharyngeal swallowing as a function of the volume and consistency of the bolus. Dysphagia.

[25] Rayneau P, Bouteloup R, Rouf C, Makris P, Moriniere S (2021). Automatic detection and analysis of swallowing sounds in healthy subjects and in patients with pharyngolaryngeal cancer. Dysphagia.

[26] Morinière S, Beutter P, Boiron M (2006). Sound component duration of healthy human pharyngoesophageal swallowing: a gender comparison study. Dysphagia.

[27] Tanaka N, Nohara K, Kotani Y, Matsumura M, Sakai T (2013). Swallowing frequency in elderly people during daily life. J Oral Rehabil.

[28] Im I, Kim Y, Oommen E, Kim H, Ko MH (2012). The effects of bolus consistency in pharyngeal transit duration during normal swallowing. Ann Rehabil Med.

[29] Pongpipatpaiboon K, Inamoto Y, Saitoh E, Kagaya H, Shibata S, Aoyagi Y (2018). Pharyngeal swallowing in older adults: Kinematic analysis using three-dimensional dynamic computed tomography. J Oral Rehabil.

[30] Seidler RD, Bernard JA, Burutolu TB, Fling BW, Gordon MT, Gwin JT (2010). Motor control and aging: links to age-related brain structural, functional, and biochemical effects. Neurosci Biobehav Rev.

[31] Christmas C, Rogus-Pulia N (2019). Swallowing disorders in the older population. J Am Geriatr Soc.

[32] Lee J, Sejdić E, Steele CM, Chau T (2010). Effects of liquid stimuli on dual-axis swallowing accelerometry signals in a healthy population. Biomed Eng Online.

[33] Taniguchi H, Tsukada T, Ootaki S, Yamada Y, Inoue M (2008). Correspondence between food consistency and suprahyoid muscle activity, tongue pressure, and bolus transit times during the oropharyngeal phase of swallowing. J Appl Physiol Bethesda Md 1985..

[34] Jaghbeer M, Sutt AL, Bergström L (2023). Dysphagia management and cervical auscultation: reliability and validity against FEES. Dysphagia.

[35] Bergström L, Cichero JA (2022). Dysphagia management: Does structured training improve the validity and reliability of cervical auscultation?. Int J Speech Lang Pathol.

[36] Makeyev O, Lopez-Meyer P, Schuckers S, Besio W, Sazonov E (2012). Automatic food intake detection based on swallowing sounds. Biomed Signal Process Control.

[37] Jayatilake D, Ueno T, Teramoto Y, Nakai K, Hidaka K, Ayuzawa S (2015). Smartphone-based real-time assessment of swallowing ability from the swallowing sound. IEEE J Transl Eng Health Med.

