# Correction to: Acoustic Analysis of Swallowing of an Experimental Meal of Three Food Textures: A Comparative Aging Study

**DOI:** 10.1007/s00455-023-10661-3

**Published:** 2024-01-10

**Authors:** Jean Baqué, Océane Huret, Pierre Rayneau, Marianne Schleich, Sylvain Morinière

**Affiliations:** 1 ENT and Head and Neck Surgery, University Hospital of Tours, 2 Boulevard Tonnelé, 37044 Tours, France; 2 Francois‐Rabelais University of Tours, University Hospital of Tours, 10 Boulevard Tonnelé, 37032 Tours, France

**Correction to: Dysphagia** 10.1007/s00455-023-10629-3

In the original version of this article, the given and family names of all authors were incorrectly structured.

Incorrect Author names: Baqué Jean, Huret Océane, Rayneau Pierre, Schleich Marianne, Morinière Sylvain.

Correct Author names: Jean Baqué, Océane Huret, Pierre Rayneau, Marianne Schleich, Sylvain Morinière.

The author group has been updated above and the original article has been corrected.

